# High-throughput dataset of impurity adsorption on common catalysts in biomass upgrading applications

**DOI:** 10.1038/s41597-024-03872-2

**Published:** 2024-09-27

**Authors:** Michelle A. Nolen, Sean A. Tacey, Martha A. Arellano-Treviño, Kurt M. Van Allsburg, Carrie A. Farberow

**Affiliations:** 1 Catalytic Carbon Transformation & Scale-Up Center, National Renewable Energy Laboratory, Golden, CO 80401 USA; 2 Department of Chemical and Biological Engineering, Colorado School of Mines, Golden, CO 80401 USA

**Keywords:** Biofuels, Heterogeneous catalysis, Atomistic models, Density functional theory, Sustainability

## Abstract

An extensive dataset consisting of adsorption energies of pernicious impurities present in biomass upgrading processes on common catalysts and support materials has been generated. This work aims to inform catalyst and process development for the conversion of biomass-derived feedstocks to fuels and chemicals. A high-throughput workflow was developed to execute density functional theory calculations for a diverse set of atomic (Al, B, Ca, Cl, Fe, K, Mg, Mn, N, Na, P, S, Si, Zn) and molecular (COS, H_2_S, HCl, HCN, K_2_O, KCl, NH_3_) species on 35 unique surfaces for transition-metal (Ag, Au, Co, Cu, Fe, Ir, Ni, Pd, Pt, Re, Rh, Ru) and metal-oxide (Al_2_O_3_, MgO, anatase-TiO_2_, rutile-TiO_2_, ZnO, ZrO_2_) catalysts and supports. Approximately 3,000 unique adsorption geometries and corresponding adsorption energies were obtained.

## Background & Summary

Biomass upgrading processes to produce bio-derived fuels and chemicals can help drive the decarbonization of historically petroleum-based industries. For example, biomass upgrading to produce drop-in biofuels can decarbonize hard-to-electrify forms of transportation, such as heavy-duty, rail, marine, and aviation vehicles^1^. Despite significant advances in developing commercially viable biomass conversion processes, these pathways still face several key challenges, including poor catalyst durability in the presence of real biomass feedstocks^1–4^. Both inorganic and organic impurities in biomass feedstocks can have deleterious effects on catalyst lifetime. Current research into mitigating the effects of these impurities is hindered by the wide range of impurities, catalysts, and supports that are utilized in biomass-upgrading chemistry and a poor understanding of the specific deactivating mechanisms^2–4^. High-throughput analyses based on density functional theory (DFT) can help address this research and development challenge, by providing information about the binding mode and strength of common biomass-derived impurities to catalysts and supports frequently used in biomass upgrading conversion processes.

**Fig. 1:**
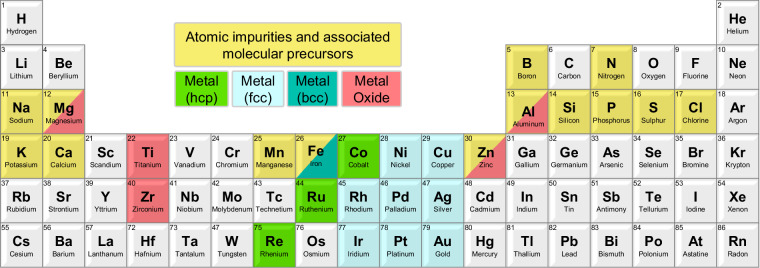
The periodic table (Groups 1–6, lanthanides omitted) highlighting the metals, metal oxides, and adsorbates explored in this study, including 12 transition-metals and five metal-oxides, with 14 atomic and seven molecular impurities. Molecular species are indicated based on constituent elements other than C, H, O.

**Fig. 2:**
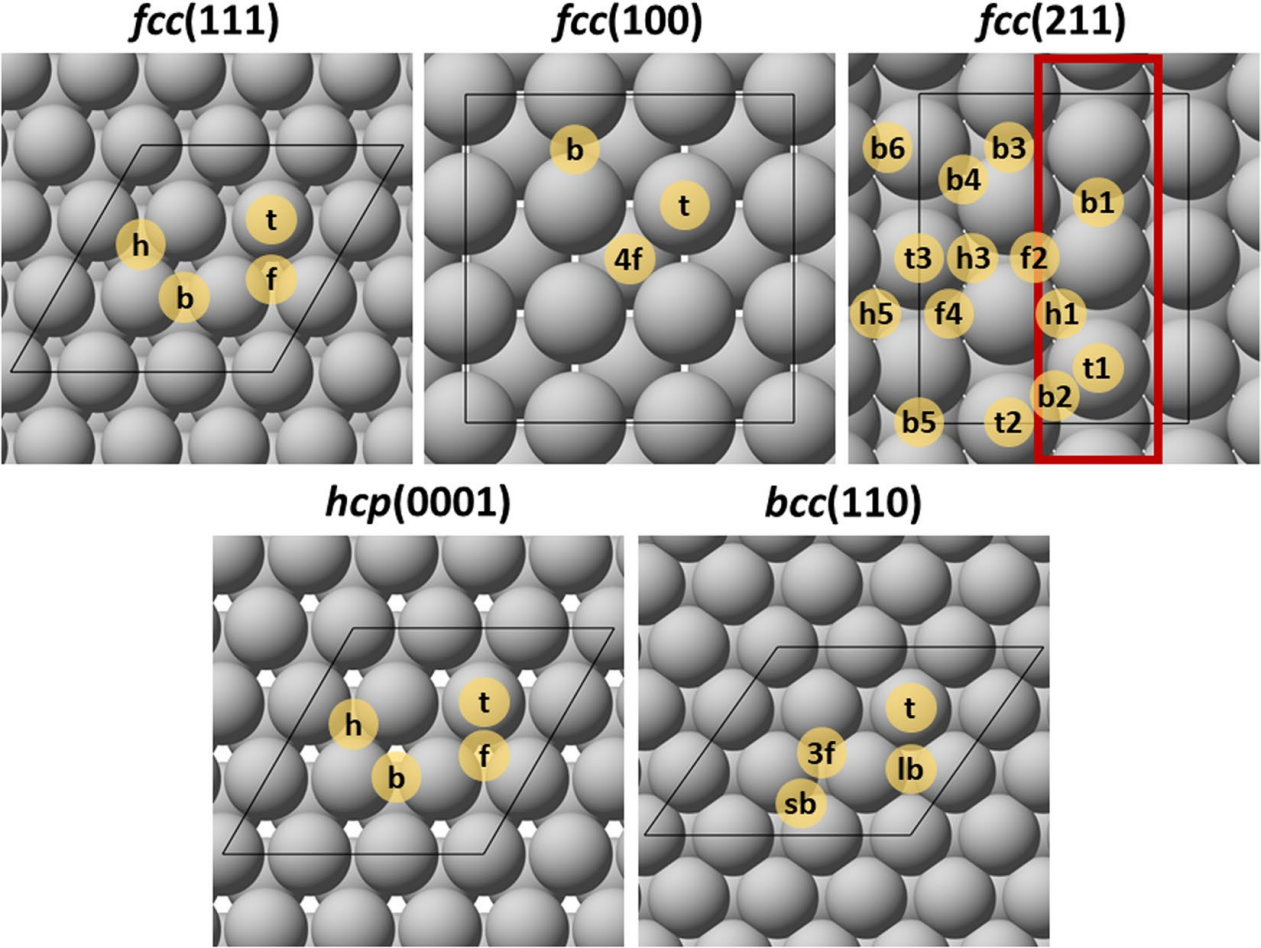
Top views of the *fcc*(111), *fcc*(100), *fcc*(211), *hcp*(0001), and *bcc*(110) surfaces with the unique high-symmetry sites labelled. Black lines denote the surface unit cell; the red lines for *fcc*(211) indicate the step edge.

**Fig. 3:**
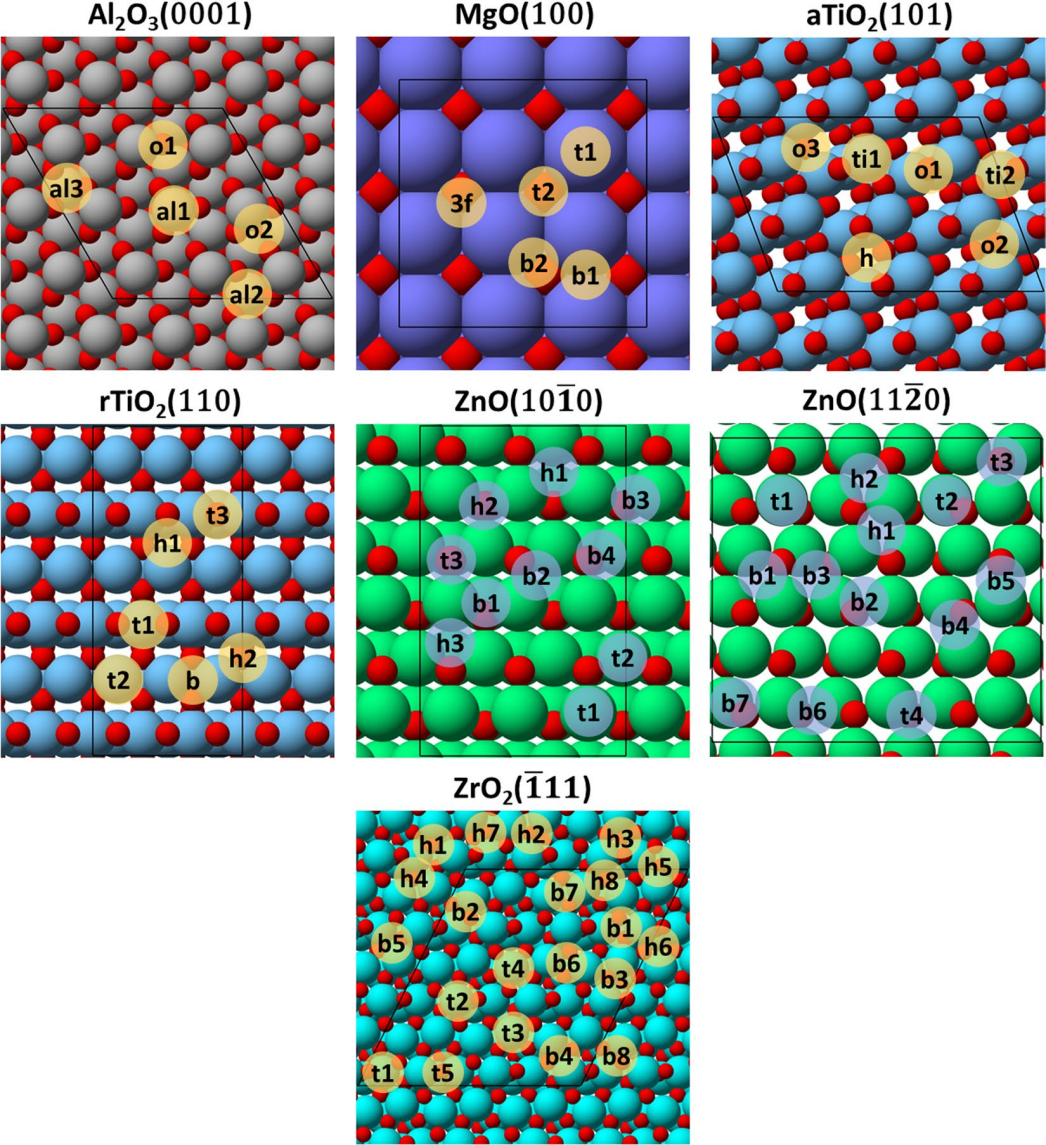
Top views of the α-Al_2_O_3_(0001), MgO(100), anatase-TiO_2_(101), rutile-TiO_2_(110), ZnO($10\bar{1}0$), ZnO($11\bar{2}0$), and ZrO_2_($\bar{1}11$) surfaces with the unique high-symmetry sites labelled. Red spheres indicate O atoms and all other sphere colors indicate the respective metal atoms. Black lines denote the surface unit cell.

This work provides an extensive DFT dataset of the adsorption of the most common and/or pernicious catalyst impurities present in biomass upgrading processes, including many that are relevant to sustainable aviation fuel (SAF) production^1,2^. Specifically, adsorption modes and energies were evaluated for fourteen atomic (Al, B, Ca, Cl, Fe, K, Mg, Mn, N, Na, P, S, Si, Zn) and seven molecular (COS, H_2_S, HCl, HCN, K_2_O, KCl, NH_3_) species on unique high-symmetry sites for a range of transition-metal and metal-oxide surfaces representing commonly used catalysts or supports (Fig. 1). Based on their known stability, close-packed transition-metal surfaces were considered including (111) facets for face centered cubic (*fcc*) metals (Ag, Au, Cu, Ir, Ni, Pd, Pt, Rh); (0001) facets for hexagonal close packed (*hcp*) metals (Co, Re, Ru); and the (110) facet for the body centered cubic (*bcc*) metal Fe^5–7^. In addition, the role of undercoordinated sites on impurity adsorption was assessed for the *fcc* metals by considering open (100) and stepped (211) surfaces (Fig. 2). For metal-oxide materials, the most-stable facets were modelled based on prior assessments of surface stability, and include α-Al_2_O_3_(0001)^8^, MgO(100)^9^, anatase-TiO_2_(101)^10^, rutile-TiO_2_(110)^11^, ZnO($10\bar{1}0$)^12,13^, ZnO($11\bar{2}0$)^12,13^, and ZrO_2_($\bar{1}11$)^14^ (Fig. 3). By evaluating the adsorption of biomass-derived impurities on a wide range of transition-metal and metal-oxide catalyst surfaces, we aim to grow the breadth of data available to the catalyst and process development research community to accelerate the pace of research through enhanced understanding of the propensity of these impurities to poison common catalysts used in biomass upgrading processes.

## Methods

**Scheme 1:**
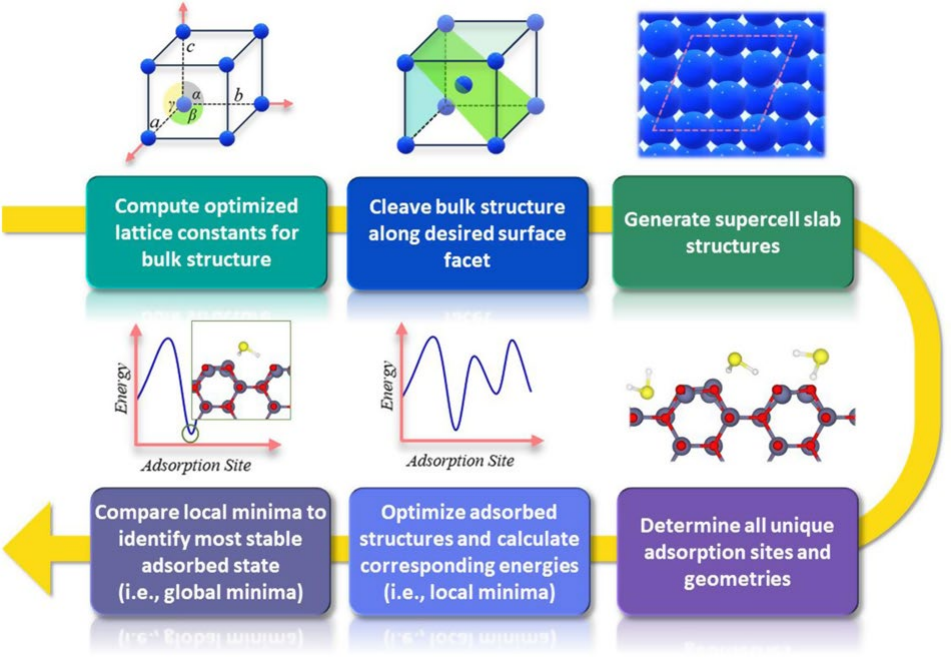
Workflow for generating optimized adsorption geometries and corresponding adsorption energies, beginning with the construction of the model surface. Approximately 9,000 initial adsorbate geometries were generated on transition-metal and metal-oxide surfaces to identify ca. 3,000 local- and global-minimum adsorption structures.

To generate the dataset, in total ca. 9,000 structure optimizations were performed to determine ca. 3,000 local- and global-minimum adsorption structures using the workflow depicted in Scheme 1. The procedure included (1) bulk material lattice optimization, (2) cleavage of the bulk structure to generate the desired surface facet, (3) generation of the slab supercell model, (4) determination of unique adsorption sites and geometries, (5) geometry optimization via DFT calculation, (6) identification of local minima states through comparison of energies corresponding to the optimized structures.

The DFT calculations were implemented using the Vienna Ab initio Simulation Package (VASP 5.4.4)^15,16^. The exchange-correlation functional was approximated using the generalized-gradient approximation (GGA) through the Perdew-Burke-Ernzerhof (PBE) exchange-correlation functional^17^. The D3 method developed by Grimme and co-workers was added to account for dispersion interactions^18^. Projector augmented-wave (PAW) potentials were used to describe electron-ion interactions^19,20^, and valence-electron wavefunctions were expanded through a plane-wave basis set with an energy cut-off of 400 eV. Spin-polarized adjustments to the GGA were implemented for all calculations. Ionic convergence occurred when the forces acting upon each atom were less than 0.02 eV/Å.

Impurity adsorption was evaluated on 12 transition metals (Ag, Au, Co, Cu, Ir, Ni, Pd, Pt, Re, Rh, and Ru) and five metal oxides (Al_2_O_3_, MgO, TiO_2_, ZnO, and ZrO_2_), selected based on their potential application as catalysts and/or supports in biomass-upgrading reactions. Unix- and Python-based scripts were developed to automate generation of input files for simulating the adsorption of impurities to each of the unique high-symmetry sites for each surface model. For the transition metals, the thermodynamically favoured close-packed surfaces were modelled with slab models composed of 4 atomic layers: (111) for *fcc* metals (Ag, Au, Cu, Ir, Ni, Pd, Pt, and Rh), (0001) for *hcp* metals (Co, Re, and Ru), and (110) for *bcc* metals (Fe). In addition, adsorption on the open (100) and stepped (211) surfaces was evaluated for the *fcc* metals. The *fcc*(111), *fcc*(100), *hcp*(0001), and *bcc*(110) surfaces were modelled through a (3 × 3) periodic surface unit cell, whereas the *fcc*(211) surfaces were simulated using a (1 × 3) periodic surface unit cell.

For the metal-oxide surfaces, the most-stable surface facets were employed for each material: α-Al_2_O_3_(0001) ((3 × 3 × 9) surface unit cell; Al-O_3_-R termination), MgO(100) ((3 × 3 × 4) surface unit cell), anatase-TiO_2_(101) ((3 × 3 × 12) surface unit cell; O-R termination), rutile-TiO_2_(110) ((3 × 3 × 8) surface unit cell; O-R termination), mixed-terminated ZnO($10\bar{1}0$) and ZnO($11\bar{2}0$) surfaces ((3 × 3 × 8) surface unit cells); and ZrO_2_($\bar{1}11$) ((3 × 3 × 24) surface unit cell; O-R termination). Stoichiometric termination sequences are specified for metal-oxide facets that can exhibit different surface terminations. For example, the α-Al_2_O_3_(0001) surface was terminated with an Al-O_3_-R sequence, where R indicates the bulk ordering of the crystal structure in the *z* direction. For anatase-TiO_2_(101) ((*U*_eff,Ti_ = 2.5 eV)), rutile-TiO_2_(110) (*U*_eff,Ti_ = 2 eV), and ZrO_2_($\bar{1}11$) (*U*_eff,Zr_ = 8 eV and *U*_eff,O_ = 4.35 eV), +*U* corrections were implemented utilizing the method by Dudarev *et al*. to account for on-site Coulombic repulsions^21^.

At least 10 Å of vacuum separated successive slabs in the *z* direction for all surface models. During geometry optimization, atoms in the top half of each surface model, including the middle layer for surfaces with an odd number of atomic layers, were allowed to fully relax while the atoms in the bottom half were fixed in their bulk-truncated positions. For the Ag and Au transition-metal surfaces, a 6 × 6 × 1 Monkhorst-Pack k-point mesh^22^ was used to sample the surface Brillouin zone, whereas a 4 × 4 × 1 Monkhorst-Pack k-point mesh was utilized for all other transition-metal surfaces as determined from convergence testing calculations. The Monkhorst-Pack k-point mesh used for the metal-oxide surfaces were: Al_2_O_3_(0001) – 4 × 4 × 1; MgO(100), anatase-TiO_2_(101), and rutile-TiO_2_(110) – 2 × 2 × 1; ZnO($10\bar{1}0$) and ZrO_2_($\bar{1}11$) – 2 × 1 × 1; and ZnO($11\bar{2}0$) – 1 × 2 × 1.

Due to their larger size, KCl and K_2_O adsorption were modelled on (4 × 4) unit cells for the *fcc*(111), *fcc*(100), *hcp*(0001), *bcc*(110), and MgO(100) surface models, and (2 × 4) unit cells for the *fcc*(211) surface models. A 4 × 4 × 1 Monkhorst-Pack k-point mesh was used for the larger (4 × 4) Ag and Au surface unit cells. For all other surfaces, a 2 × 2 × 1 Monkhorst-Pack k-point mesh was used for K_2_O and KCl adsorption.

Adsorption on each surface model was limited to only one exposed side of the slab, with the required dipole correction applied to the electrostatic potential^23^. The adsorption strength of each impurity was quantified by its binding energy (*E*_B_), defined as:

1 $${E}_{{\rm{B}}}={E}_{{\rm{t}}{\rm{o}}{\rm{t}}}\,\mbox{--}\,{E}_{{\rm{c}}{\rm{l}}{\rm{e}}{\rm{a}}{\rm{n}}}\,\mbox{--}\,{E}_{{\rm{g}}{\rm{a}}{\rm{s}}}$$

where *E*_tot_, *E*_clean_, and *E*_gas_ are the total energy of the adsorbate + surface complex, clean surface, and adsorbate in the gas-phase, respectively. By this definition, a more negative binding energy corresponds to stronger adsorption and thus the possibility of a higher propensity for catalyst poisoning.

**Fig. 4:**
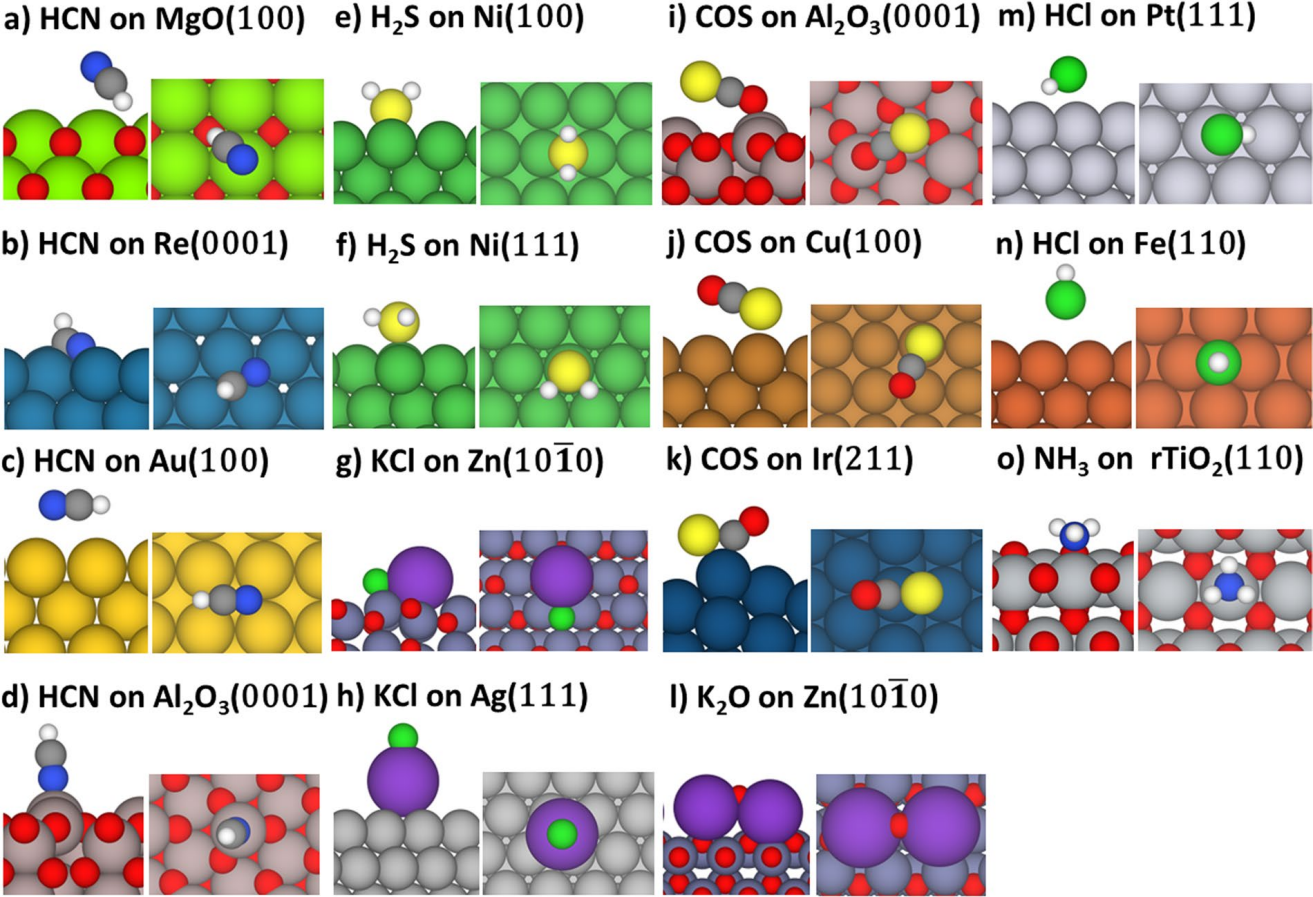
Representative snapshots of unique adsorption geometries of the studied molecular species ((**a**-**d**) HCN, (**e**-**f**) H_2_S, (**g**-**h**) KCl, (**i**-**k**) COS, (**l**) K_2_O, (**m**-**n**) HCl, (**o**) NH_3_) on transition-metal and metal-oxide surfaces.

**Fig. 5:**
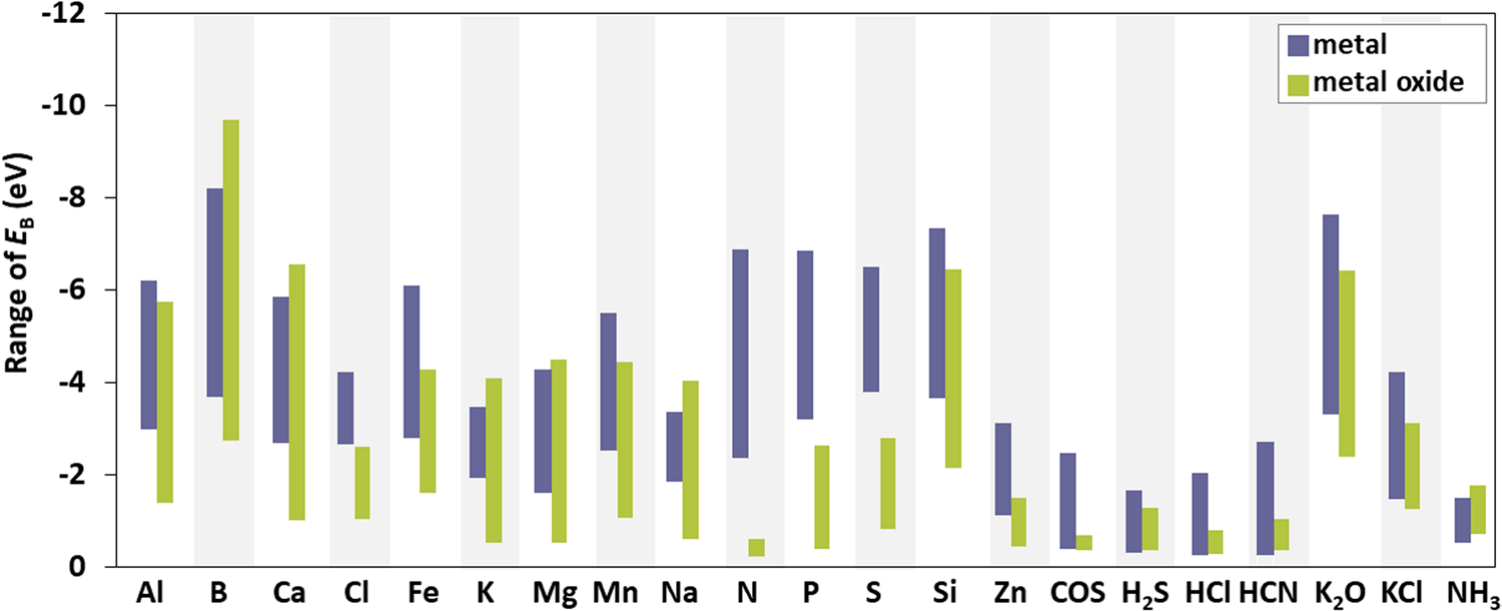
Range of calculated binding energies (*E*_B_, ranging from largest to smallest) of atomic and molecular species on the studied metal and metal oxide surfaces. A more negative binding energy indicates more favorable adsorption.

Only non-dissociative (molecular) adsorption configurations of molecular species were reported. The DDEC6 (density derived electrostatic and chemical)^24^ bond orders were calculated to distinguish elongated vs. dissociated intramolecular bonds of adsorbates; dissociatively adsorbed species were defined as having >75% reduction of intramolecular bond order from gas-phase values. Adsorption geometries of COS, K_2_O, and HCN were generated roughly parallel to the metal and metal-oxide surfaces. For HCl and KCl adsorption, both perpendicular and parallel adsorption geometries were identified. For H_2_S and NH_3_, geometries were generated for adsorption through S and N atoms, respectively. Structures were visualized with Atomic Simulation Environment (ASE) 3.22.1^25^. Representative examples of converged geometries generated with Visualization for Electronic and Structure Analysis (VESTA)^26^ are depicted in Fig. 4. The range of adsorption energies computed for each impurity across the metal or metal-oxide surfaces evaluated are reported in Fig. 5.

## Data Records

The converged adsorption structure (CONTCAR) and output files (OUTCAR) for each unique adsorption geometry and a JSON file of the complete, parsed dataset in the ChemCatBio Catalyst Property Database (CPD)^27^ data structure are provided in the Materials Cloud archive^28^. All unique local-minimum adsorption geometries for each adsorbate on each surface are included, and the global-minimum adsorption geometry on each surface is indicated in JSON via ‘is_most_stable_site.’ The Materials Cloud archive file structure for the structure and output files is organized hierarchically, beginning with surface (titled as *surface_facet*; e.g., Ag_100), followed by subdirectories for each atomic or molecular adsorbate (e.g., Al, HCl, N, etc.). For molecular species COS, HCl, HCN, K_2_O, and KCl, an additional subdirectory specifies the orientation of the bound molecule (diagonal (diag), left-right (lr), up-down (ud), side (side), or vertical (vert)). For all other adsorbates, subdirectories only specify the adsorption site of the adsorbate (e.g., top (t), bridge (b)) according to the site assignments defined in Figs. 1 and 2. A file detailing the directory structure is provided within the Materials Cloud Archive (README.txt). The output files can be viewed and adapted using any text editor and visualized using any common atomistic visualization software, such as ASE^25^ or VESTA^26^. The complete dataset, including all metadata, can also be searched and filtered and the results downloaded as a JSON within the ChemCatBio CPD.

## Technical Validation

All bulk structures were validated prior to simulating adsorption of the impurity species via comparison of the optimized, computed bulk lattice constants with experimental literature values. The corresponding lattice constant values for metals (Table 1) and metal oxides (Table 2) show good agreement for all materials.

**Table 1:** Calculated and experimental lattice parameters for metals.

Metal	Calculated		Experimental	
	*a* (Å)	*c* (Å)	*a* (Å)	*c* (Å)
Ag	4.074	—	4.078^29^	—
Au	4.099	—	4.0786^30^	—
Co	2.470	3.983	2.503^31^	4.0574^31^
Cu	3.567	—	3.61^32^	—
Fe	2.801	—	2.866^33^	—
Ir	3.837	—	3.8392^34^	—
Ni	3.479	—	3.524^35^	—
Pd	3.885	—	3.8909^36^	—
Pt	3.920	—	3.924^37^	—
Re	2.752	4.463	2.762^38^	4.455^38^
Rh	3.786	—	3.8^39^	—
Ru	2.692	4.248	2.7062^40^	4.2815^40^

**Table 2:** Calculated and experimental lattice parameters for metal-oxides.

Metal Oxide	Calculated				Experimental			
	*a* (Å)	*b* (Å)	*c* (Å)	β (°)	*a* (Å)	*b* (Å)	*c* (Å)	β (°)
MgO	4.21	—	—	—	4.217^41^	—	—	—
ZnO	3.283	—	5.260	—	3.258^42^	—	5.22^43^	—
Al_2_O_3_	4.789	—	13.063	—	4.7591^44^	—	12.9894^43^	—
ZrO_2_	5.277	5.341	5.447	99.470	5.146^43^	5.205^43^	5.313^43^	99.1^43^
aTiO_2_	3.865	—	9.627	—	3.782^45^	—	9.50226^45^	—
rTiO_2_	4.664	—	2.996	—	4.587^45^	—	2.954^45^	—

Adsorbed structures, including a retained molecular state for all molecular species, were confirmed via visualization of the corresponding structures and calculations of their respective bond orders as described in the Methods section. When available, the calculated adsorption energies are compared with previously reported values in the literature computed with identical or very similar (i.e., identical exchange-correlation functional and potentials) methods (Table 3). Comparable literature data for validation was identified for the more commonly studied atomic and molecular impurities, including S, H_2_S, N, NH_3_, and K. In most cases, the adsorption energies calculated in this work are within ±0.20 eV of the previously reported values. In cases where the values differ by more than 0.20 eV, including the largest discrepancy identified across the dataset (−0.45 eV) for H_2_S adsorption on Cu(111), the difference can likely be attributed to the inclusion of dispersion interactions in the calculations reported here resulting in a stronger predicted binding energy compared to the prior report. Additional differences between this work and data in the literature may be attributed to (i) inclusion of zero-point energy (ZPE) corrections in some other works, as ZPE corrections were not included in the dataset reported here and/or (ii) variations in surface coverage. Toward the latter, the comparison data obtained from the literature was limited to simulations at the clean-surface limit (i.e., sufficiently low coverage that lateral interactions should be insignificant), however we note that in some cases the specific coverage simulated was not clearly defined in the cited reference.

**Table 3:** Comparison of adsorption energies calculated in this work (Calc.) and reported in the literature (Lit.) for a sample of the more commonly studied atomic and molecular impurities on transition-metal and metal-oxide surfaces at the clean-surface limit.

Metal	S		H_2_S		N		NH_3_		K	
	Calc.	Lit.	Calc.	Lit.	Calc.	Lit.	Calc.	Lit.	Calc.	Lit.
Ag(111)	−3.87	−3.64^46^^,a^	−0.46	−0.17^47^^,a^	−2.37	−2.34^48^^,b^	−0.53	−0.55^48^^,b^	−2.07	−1.96^49^^,a^
Au(111)	−3.78	−3.69^46^^,a^	−0.31	−0.27^47^^,a^	−2.69	−2.6^48^^,b^	−0.59	−0.61^48^^,b^	−2.61	−2.7^50^^,a^
Co(0001)	−5.43	−5.54^51^^,b,c^	—	—	−5.77	−5.36^48^^,b^	−0.97	−0.93^48^^,b^	—	—
Cu(111)	−4.71	−4.40^46^^,a^	−0.71	−0.26^47^^,a^	−3.93	−3.98^48^^,b^	−0.82	−0.75^48^^,b^	−2.46	−2.35^52^^,a^
Fe(110)	−5.92	−5.91^53^	—	—	−6.64	−6.8^48^^,b^	−0.96	−0.96^48^^,b^	—	—
Ir(111)	−5.56	−5.57^47^^,a^	−1.13	−0.77^47^^,a^	—	—	—	—	—	—
Ni(111)	−5.45	−5.27^54^^,a^	−0.99	0.56^47^^,a^	−5.43	−5.36^48^^,b^	−1.05	−0.97^48^^,b^	—	—
Pd(111)	—	—	−1.08	−1.12^55^	−4.94	−4.79^48^^,b^	−0.98	−1.02^48^^,b^	—	—
Pt(111)	−5.32	−5.23^54^^,a^	−1.23	−0.91^47^^,a^	—	—	—	—	—	—
Re(0001)	−5.89	−5.98^54^^,a^	—	—	—	—	—	—	—	—
Rh(111)	−5.74	−5.50^54^^,a^	—	—	—	—	—	—	—	—
Ru(0001)	−5.96	−5.75^54^^,a^	—	—	−6.37	−6.438^56^	—	—	—	—
Al_2_O_3_(0001)	−1.32	−1.05^57^^,a^	—	—		—	—	—	—	—
aTiO_2_(101)	—	—	—	—	—	—	−1.40	−1.16^58^^,a^	—	—

^a^No dispersion corrections.

^b^PBE-D2 method and ZPE corrections applied.

^c^Pseudopotentials not specified.

## Data Availability

No custom code is used.
